# The truncated major pilin subunit SBP2’ contributes to *Streptococcus suis* meningitis by interacting with host plasminogen

**DOI:** 10.1128/aem.01267-26

**Published:** 2026-08-10

**Authors:** Genglin Guo, Pei Li, Yu Zhou, Quan Li, Yanfei Yu, Jiahui An, Wei Zhang

**Affiliations:** 1 Department of Microbiology and Immunology, Drexel University College of Medicine 12312, Philadelphia, Pennsylvania, USA; 2 Jiaodong Innovation Center, Shandong Institute of Sericulture, Shandong Academy of Agricultural Sciences 74641, Yantai, China; 3 College of Veterinary Medicine, Nanjing Agricultural University 70578, Nanjing, China; 4 The Sanya Institute of Nanjing Agricultural University, Sanya, China; 5 College of Life Sciences and Food Engineering, Hebei University of Engineering 117798, Handan, China; 6 College of Veterinary Medicine, Yangzhou University 38043, Yangzhou, China; 7 Key Laboratory of Veterinary Biological Engineering and Technology, Institute of Veterinary Medicine, Jiangsu Academy of Agricultural Sciences, Ministry of Agriculture and Rural Affairs 117941, Nanjing, China; 8 Department of Microbiology, Immunology and Inflammation, Center for Inflammation and Lung Research, Temple University Lewis Katz School of Medicine 12314, Philadelphia, Pennsylvania, USA; Universite de la Reunion, Ste Clotilde, France

**Keywords:** *Streptococcus suis*, pili, SBP2’, virulence, plasminogen, meningitis, monoclonal antibody

## Abstract

Meningitis is one of the most striking manifestations of *Streptococcus suis* infection. Although multiple virulence-associated factors have been identified, the mechanisms underlying meningitis development remain incompletely understood. In *S. suis*, a *srtBCD* pilus gene cluster has been identified; our previous study suggested its association with bacterial virulence. In this study, we investigated the role of the *srtBCD* cluster in *S. suis* pathogenesis and confirmed that SBP2’ was specifically localized on the bacterial surface and was found to be essential for full virulence in a murine infection model, whereas deletion of other minor subunits had minimal impact. Using brain microvascular endothelial cells (BMECs), microglia, and a BALB/c mouse model, we examined how SBP2’ promotes the development of *S. suis* meningitis. Our results show that SBP2’ enhances bacterial colonization of BMECs and recruits host plasminogen, facilitating extracellular matrix (ECM) degradation, and thereby promoting bacterial invasion. In addition, SBP2’ enables *S. suis* to evade microglial phagocytosis and improves its intracellular survival. Importantly, a monoclonal antibody targeting SBP2’ conferred significant immunoprotection in mice. Together, these findings identify SBP2’ as an important virulence factor and plasminogen-binding receptor that facilitates *S. suis* traversal of the blood-brain barrier.

**IMPORTANCE:**

*Streptococcus suis* is an important zoonotic bacterial pathogen that could lead to severe damage in the central nervous system, but the mechanism underlying the breakthrough of the blood-brain barrier is still unclear. Pili are commonly considered virulence factors and subunit vaccine candidates, but no pilus structure could be observed on the surface of *S. suis* serotype 2. In this study, we found that the *srtBCD* pilus cluster, which was considered a pseudogene because of the truncation of the major pilus subunit, could be expressed, and the truncated major pilus subunit could be detected on the cell surface and be involved in the pathogenesis of *S. suis* serotype 2. Notably, monoclonal antibodies targeting SBP2’ provide immunoprotection in mice, reducing bacterial loads and brain damage. These findings identify SBP2’ as a key virulence factor and therapeutic target, offering insights into *S. suis* meningitis mechanisms.

## INTRODUCTION

*Streptococcus suis* (*S. suis*) is an emerging zoonotic pathogen that poses a substantial threat to both the swine industry and public health (1, 2). Among the 29 recognized classic serotypes of *S. suis*, serotype 2 is the most prevalent, virulent, and predominant serotype responsible for human infections (3). In addition to serotype classification, multilocus sequence typing (MLST) has further revealed that genetic background plays a crucial role in *S. suis* virulence. Notably, most highly virulent serotype 2 strains associated with invasive human infections, particularly meningitis cases, belong to clonal complex 1 (CC1). In contrast, many avirulent serotype 2 isolates are more frequently assigned to other CCs like CC28, which are commonly associated with asymptomatic carriage or limited pathogenic potential (4). Since the first human infection case was reported in Denmark, *S. suis* infections have been documented in more than 30 countries and regions worldwide (5). In China, two major outbreaks occurred in Jiangsu in 1998 and Sichuan in 2005, resulting in 14 and 38 deaths, respectively, and drawing significant public attention due to their severe public health impact (6). Over the past 40 years, more than 100 virulence-associated factors have been identified in *S. suis* (7). Among them, capsular polysaccharide (CPS), muramidase-released protein (MRP), extracellular factor (EF), and suilysin (SLY) are considered the most critical determinants of virulence (8). However, isolates lacking these classical virulence markers have also been reported to cause disease (9), suggesting that *S. suis* pathogenesis is multifactorial and more complex than previously assumed.

Meningitis is one of the most prominent clinical manifestations of *S. suis* infection and is characterized by the presence of fibrin deposition, edema, and cellular infiltration in the meninges and choroid plexus (10). Numerous studies have focused on the mechanisms by which *S. suis* breaches the blood-brain barrier (BBB), and several proteins—such as MRP (11), Enolase (12), and SspepO (13)—have been identified as being associated with meningitis development. The BBB is a highly complex structure composed of multiple cell types and tissue components. Although brain microvascular endothelial cells (BMECs) are commonly used to model BBB penetration, astrocytes and microglia have recently been recognized as additional relevant cell models for studying central nervous system infection, particularly in the context of inflammatory responses (14). Notably, microglia appear to be more strongly associated with the expression of pro-inflammatory genes compared with astrocytes (15). It is generally postulated that colonization of the BBB and subsequent penetration constitute the initial steps in *S. suis*–induced BBB disruption, and that endothelial injury, bacterial transcytosis, and inflammation together contribute to the eventual development of meningitis.

Pili are widely recognized as important factors involved in brain infection and cross-species transmission in many bacterial pathogens, such as *Neisseria meningitidis*, *Streptococcus pneumoniae*, *Salmonella spp*. (16–18). In 2009, a pilus gene cluster termed *srtBCD*, which is homologous to the *S. pneumoniae rlrA* pilus gene cluster, was identified in the *S. suis* genome (19). This cluster was long regarded as a pseudogene set and largely overlooked due to the truncation of the major pilin subunit SBP2. However, our group later demonstrated that the truncated pilin subunit SBP2’ is indeed expressed and plays an important role in *S. suis* virulence and infection processes (20–22). Furthermore, a genome-wide association study revealed that *srtBCD* may serve as an important virulence-associated marker in *S. suis* serotype 2 (23), with a distribution pattern that appears to be closely linked to clonal complex structure. Notably, *srtBCD* is predominantly present in highly virulent lineages, particularly clonal complex 1 (CC1), which is strongly associated with invasive human infections, whereas it is largely absent in low-virulence lineages such as CC28 and other related clonal complexes that are mainly associated with asymptomatic carriage. This lineage-specific distribution further supports the contribution of genetic background to the differential virulence potential of *S. suis* (21).

The recruitment of plasminogen to the bacterial cell surface has emerged as a key feature of host-pathogen interactions. Plasminogen, a glycoprotein present in plasma and extracellular fluids at concentrations of approximately 2 μM, can be converted into the serine protease plasmin upon activation. Plasmin possesses broad proteolytic activity and is capable of degrading fibrin clots, connective tissue, and components of the extracellular matrix (ECM) (24). The ECM—secreted by cells in nearly all mammalian tissues and organs—has been reported to play an essential role in mediating pathogen adherence to host cells (25, 26). Plasminogen receptors are typically surface-exposed proteins; a well-known example in *Streptococci* is the M protein of Group A *Streptococcus* (27). In *S. suis*, several surface proteins have also been identified as plasminogen receptors, including MRP (11) and SspepO (13), as we mentioned above. In this study, we aimed to further investigate the role of SBP2’ in *S. suis* meningitis, with a particular focus on how SBP2’ interacts with host molecules such as plasminogen and contributes to the bacterium’s ability to penetrate the blood-brain barrier.

## MATERIALS AND METHODS

### Bacterial strains and cell culture

The virulent serotype 2 (*S. suis* SS2) strain ZY05719 (ST7) was isolated from a diseased pig during the 2005 *S. suis* outbreak in Sichuan, China. Bacterial cultures were grown in Todd–Hewitt broth (THB; Becton Dickinson, USA). Human brain microvascular endothelial cells (hBMECs) were obtained from ScienCell Research Laboratories (Catalog #1000), and BV2 microglial cells were procured from the Cell Resource Center (Beijing, China). Cells were maintained in Dulbecco’s modified Eagle medium (DMEM; Gibco) supplemented with 10% fetal bovine serum (FBS; Gibco), 100 U/mL penicillin, and 10 μg/mL streptomycin. Details of bacterial isolates and primers used in this study are provided in Table S1.

### Sequence alignment

The sequences of the *srtBCD* cluster from *S. suis* strain ZY05719 and the *rlrA* pilus cluster from *S. pneumoniae* TIGR4 were extracted from the published complete genome sequences deposited in the NCBI database under accession numbers NZ_CP007497.1 and NZ_CP089948.1, respectively. Amino acid sequences were aligned using MEGA X and visualized with ESPript 3.0 (28). Secondary structure predictions were assigned based on the available structural data for *rlrA* proteins deposited in the Protein Data Bank (PDB entry codes 2Y1V, 4OQ1, 2WW8, 2WTS, 3G69, and 2W1K).

### Construction of the deletion mutants

One deletion mutant of the pilus subunit gene sbp1 (*Δsbp1*) was constructed in our previous study (22), and the other three mutants (*Δsbp2’*, *Δsbp3*, and *Δsbp4*) were constructed as follows. Deletion mutants of pilus subunit genes were constructed using the thermosensitive shuttle vector pSET4s (29). Briefly, genomic DNA of *S. suis* strain ZY05719 was extracted using the FastPure Bacteria DNA Isolation Mini Kit (Vazyme, China, Cat No. DC103-01) following the manufacturer’s instructions. The upstream and downstream flanking regions of the target genes were amplified from the extracted DNA. The upstream fragment was amplified using primer pair A/B, and the downstream fragment using primer pair C/D. These homologous arms were cloned into pSET4s to generate the recombinant plasmids. The recombinant plasmid was then electroporated into competent ZY05719 cells. Gene replacement was induced by temperature-mediated allelic exchange, and loss of the plasmid was achieved through serial passaging. Successful construction of mutants was confirmed by diagnostic PCR and Sanger sequencing. For PCR verification, primer pairs X/Y (external primers) and E/F (internal primers) were used. The *sbp2’’* mutant was not prepared in this study because our previous study demonstrated that deletion of *sbp2’’* did not significantly affect the virulence or adherence ability of *S. suis*.

### Preparation of recombinant proteins and antibodies

Recombinant proteins SBP1, SBP2’, SBP3, and SBP4 were expressed in *Escherichia coli* using the pET-32a(+) or pET-21a(+) expression vectors. SBP1, SBP3, and SBP4 were expressed using pET-32a(+), while SBP2’ was expressed using pET-21a(+) to minimize potential interference from fusion tags in subsequent monoclonal antibody production.

Proteins were purified by Ni–NTA affinity chromatography with HisTrap HP columns (GE Healthcare), followed by further purification through anion-exchange chromatography (Resource Q column) and size-exclusion chromatography (Superdex 200 Increase 10/300 GL column).

Polyclonal antiserum against rSBP1, rSBP2’, rSBP3, and rSBP4 was generated in New Zealand White rabbits. Rabbits were immunized three times at two-week intervals with 500 μg of purified rSBP2’ protein emulsified 1:1 with Montanide ISA 201 adjuvant (Seppic, France). Sera were collected and used for downstream analyses.

Further, monoclonal antibodies were produced for rSBP2’. Eight-week-old BALB/c mice were immunized with 50 μg of recombinant SBP2’ (rSBP2’) emulsified 1:1 with Montanide ISA 201 adjuvant (Seppic, France) three times at two-week intervals. Splenocytes were fused with SP2/0 myeloma cells, and hybridoma selection was performed in HAT medium. Positive clones were screened by in-house indirect ELISA, in which 96-well plates were coated with recombinant SBP2’ protein, followed by incubation with hybridoma supernatants and HRP-conjugated secondary antibodies. Signals were detected using TMB substrate at 450 nm. Monoclonal antibodies were subsequently purified via Protein G affinity chromatography performed by GenScript Co., Ltd (China). The isotypes of the monoclonal antibodies were determined using a Mouse Monoclonal Antibody Isotype ELISA Kit (Proteintech, USA).

### Subcellular localization of SBP2’

#### Flow cytometry analysis

Flow cytometry was used to analyze the surface localization of all pilus subunits. Bacteria were cultured to mid-log phase, harvested by centrifugation, and washed with PBS. Cells were then blocked with 5% bovine serum albumin (BSA) and incubated with polyclonal antiserum raised against recombinant pilus subunit proteins. PBS was used as a negative control. After washing, samples were incubated with goat anti-rabbit IgG conjugated with FITC (Boster) as the secondary antibody. Fluorescence signals were detected using a BD FACSCalibur flow cytometer and analyzed using standard acquisition settings.

#### Indirect immunofluorescence assay

For immunofluorescence analysis, bacterial cells were fixed with 4% paraformaldehyde and immobilized on glass dishes. After blocking with 5% BSA, cells were incubated with the same polyclonal antisera against recombinant pilus subunit proteins, followed by incubation with goat anti-rabbit IgG-FITC secondary antibody. Nuclei were counterstained with DAPI. Fluorescence images were acquired using a fluorescence microscope under standard FITC and DAPI filter sets.

### Animal experiments

Four-week-old BALB/c mice were used as a pathological model to evaluate the differences in virulence between the wild-type (WT) strain and the deletion mutants of *S. suis*. Mice were randomly divided into six groups (*n* = 10 per group), including the WT group, the *Δsbp1*, *Δsbp2’*, *Δsbp3*, and *Δsbp4* mutant groups, and a PBS-treated negative control group.

Each mouse was challenged intraperitoneally with 2 × 10⁸ CFU of *S. suis* in PBS and monitored for 7 days to evaluate survival. Mice in the negative control group received sterile PBS only. In accordance with animal ethics guidelines, mice that became unresponsive or recumbent were considered moribund and euthanized.

For bacterial burden analysis, mice were randomly divided into two groups (*n* = 6 per group), including the WT group and the *Δsbp2’* mutant group. Each mouse was challenged intraperitoneally with 2 × 10⁸ CFU of *S. suis* in PBS. Brains, lungs, spleens, and blood were collected after symptom onset. Tissue samples were homogenized in sterile PBS, serially diluted in 10-fold steps, and plated onto Todd–Hewitt Agar (THA) for bacterial load enumeration. Colony-forming units (CFU) were calculated after incubation. To evaluate pathological changes in the brain, hematoxylin–eosin (H&E) staining was performed.

### Far-Western blotting

Far-Western blotting was performed to evaluate the binding of rSBP2’ to host plasminogen (plg) and fibronectin (FN). Purified rSBP2’ (5 μg) was separated by SDS–PAGE and transferred onto PVDF membranes. Recombinant HP07325 (5 μg), a cytoplasmic protein previously shown not to interact with extracellular host proteins, was included as a negative control (30). After transfer, membranes were blocked with PBST containing 5% BSA to prevent nonspecific binding and incubated overnight at 4°C with human plasminogen (Sigma-Aldrich, Cat No. SRP6518) or fibronectin (Sigma-Aldrich, Cat No. 86088-83-7) at a final concentration of 5 μg/mL. After washing, membranes were sequentially incubated with rabbit anti-plasminogen antibody (Sigma-Aldrich, Cat No. HPA021602, 1:2000 dilution) or rabbit anti-fibronectin antibody (Sigma-Aldrich, Cat No. F3648, 1:2000 dilution), and HRP-conjugated goat anti-rabbit IgG antibody (Boster, Cat No. BA1055, 1:2000 dilution). Chemiluminescent signals were detected using an ECL reagent (Vazyme, China).

### ELISA-based direct binding assay

Purified rSBP2’, plasminogen, or fibronectin were used in ELISA-based direct binding assays as previously described with minor modifications (31). Briefly, 96-well microtiter plates were coated overnight at 4 °C with 100 μL of purified protein at a concentration of 5 μg/mL. After washing, wells were blocked with 5% BSA for 2 h at room temperature. Increasing concentrations of soluble plasminogen or fibronectin were then added to wells coated with immobilized rSBP2’. Reciprocal binding assays were also performed using immobilized plasminogen or fibronectin incubated with increasing concentrations of soluble rSBP2’. BSA at the same concentration was used as a negative control. After incubation and washing, bound proteins were detected using the corresponding primary antibodies followed by HRP-conjugated secondary antibodies. The absorbance was measured at 450 nm using a microplate reader.

### Surface plasmon resonance

Surface plasmon resonance (SPR) was performed using a Biacore X100 instrument to dynamically evaluate the interaction between rSBP2’ and host plasminogen, as previously described with minor modifications (32). Briefly, human plasminogen was diluted to 10 μg/mL in 10 mM sodium acetate buffer (pH 4.0) and covalently immobilized onto a CM5 sensor chip via amine coupling. Binding kinetics were analyzed using serial concentrations of rSBP2’ (1.5625, 3.125, 6.25, 12.5, and 25 μg/mL) prepared in HBS-EP running buffer containing 10 mM HEPES, 150 mM NaCl, 3 mM EDTA, and 0.05% surfactant P20 (Biacore AB). Samples were injected over the immobilized plasminogen surface at a flow rate of 30 μL/min for 180 s at 20°C. The immobilized plasminogen generated a resonance unit (RU) value of approximately 600. The dissociation phase was monitored for 1000 s by flowing running buffer over the chip. Binding kinetics were analyzed using Biacore X100 Control Software.

### Indirect immunofluorescence assay (IFA)

The interaction between bacterial proteins and host cells was assessed by immunofluorescence according to previously described methods (20). Cells were cultured to confluence on 15 mm glass-bottom culture dishes (NEST), fixed with formaldehyde, and incubated with the corresponding proteins or antibodies. The following reagents were used: monoclonal antibody against SBP2’, fibronectin (5 μg/mL; Sigma, 86088-83-7), rabbit anti-fibronectin antibody (1:2000; Sigma-Aldrich, F3648), FITC-conjugated goat anti-mouse IgG (H + L) (1:50; Boster, BA1101), and CY3-conjugated goat anti-rabbit IgG (H + L) (1:50; Boster, BA1031). Fluorescence images were acquired using a ZEISS fluorescence microscope.

### Cell adhesion and invasion assay

Human brain microvascular endothelial cells (hBMECs) were used to evaluate the adhesion and invasion abilities of *S. suis*. Cells were cultured to confluence in 24-well plates and washed with PBS before bacterial infection. For the adhesion assay, *S. suis* was added at a multiplicity of infection (MOI) of 20:1 and incubated at 37°C for 2 h. After incubation, the supernatant was removed, cells were lysed with ddH₂O, and adherent bacteria were enumerated by the plate count method. For the invasion assay, *S. suis* was added at the same MOI (20:1) and incubated with hBMECs for 1 h before treatment with 100 μg/mL gentamicin and 5 μg/mL penicillin to eliminate extracellular bacteria. Intracellular bacteria were then quantified as described above.

### ECM degradation assay

The ECM degradation assay was performed as previously described (33). Briefly, 20 μL of 1.1 μm polystyrene beads (Sigma-Aldrich, Cat No. LB11) were conjugated overnight at 4°C with rSBP2’ at a concentration of 1.5 mg/mL in a total volume of 1 mL. After washing, the beads were incubated with plasminogen at a final concentration of 10 μg/mL at 37°C for 3 h, followed by the addition of tissue-type plasminogen activator (tPA; Sigma-Aldrich, Cat No. T0831) to a final concentration of 200 ng/mL. Following treatment, the beads were resuspended in 1 mL PBS for subsequent experiments.

ECM degradation was evaluated using Matrigel (Corning, USA). Matrigel was layered onto the upper chamber of a 3 μm Transwell insert (Corning, USA) and incubated at 37°C overnight to simulate the ECM structure. Seventy microliters of the treated beads were added to the upper chamber, and 700 μL PBS was added to the lower chamber. The system was incubated at 37°C for 40 h. Transwell membranes were then gently washed with PBS, fixed with 2.5% glutaraldehyde, subjected to ethanol gradient dehydration, coated with metallic medium, and examined under a Zeiss EVO-LS10 scanning electron microscope (Zeiss, Germany).

### Phagocytosis and intracellular survival assays

The anti-phagocytic capacity of *S. suis* in BV2 microglial cells was assessed as previously described (34). Briefly, cells were cultured to confluence in 24-well plates and washed with PBS before infection. *S. suis* was added at a MOI of 50:1 and incubated at 37°C for 1 h. Extracellular bacteria were then killed using 100 μg/mL gentamicin and 5 μg/mL penicillin, and this time point was designated as 0 h. Intracellular bacteria were released by cell lysis with ddH₂O and enumerated at 1, 2, 3, and 4 h post-treatment using the plate count method.

### Passive immunization with SBP2’ monoclonal antibody

To evaluate the protective efficacy of the SBP2’ monoclonal antibody against *S. suis* infection, a passive immunization assay was performed using ICR mice according to previously described protocols (35). Mice were randomly divided into two groups (*n* = 10 per group), including the PBS-treated control group and the passive immunization group. Each mouse was administered 200 μg of anti-rSBP2’ monoclonal antibody or PBS via intraperitoneal injection 6 h prior to bacteria challenge. Mice were subsequently challenged intraperitoneally with 2 × 10⁸ CFU of WT *S. suis* strain ZY05719 and monitored for 7 days. Mice that became unresponsive or recumbent were considered moribund and euthanized in accordance with animal ethics guidelines.

### Statistical analysis

Statistical analyses and data visualization were performed using GraphPad Prism 7 software. The choice of statistical test was determined by the experimental design and data distribution. For the comparison of survival curves in mouse models, the log-rank (Mantel-Cox) test was applied. For comparisons between two experimental groups (such as ELISA, cell adhesion, and invasion assays), an unpaired, two-tailed Student’s *t*-test was used. For multi-group comparisons involving more than two groups, one-way analysis of variance (ANOVA) followed by Dunnett’s post-hoc test was performed. A *P* value of less than 0.05 was considered statistically significant.

## RESULTS

### Basic features of the *srtBCD* pilus gene cluster

A pilus gene cluster homologous to the *rlrA* pilus cluster in *S. pneumoniae* was identified in *S. suis* serotype 2 and designated the *srtBCD* pilus gene cluster.

**Fig 1:**
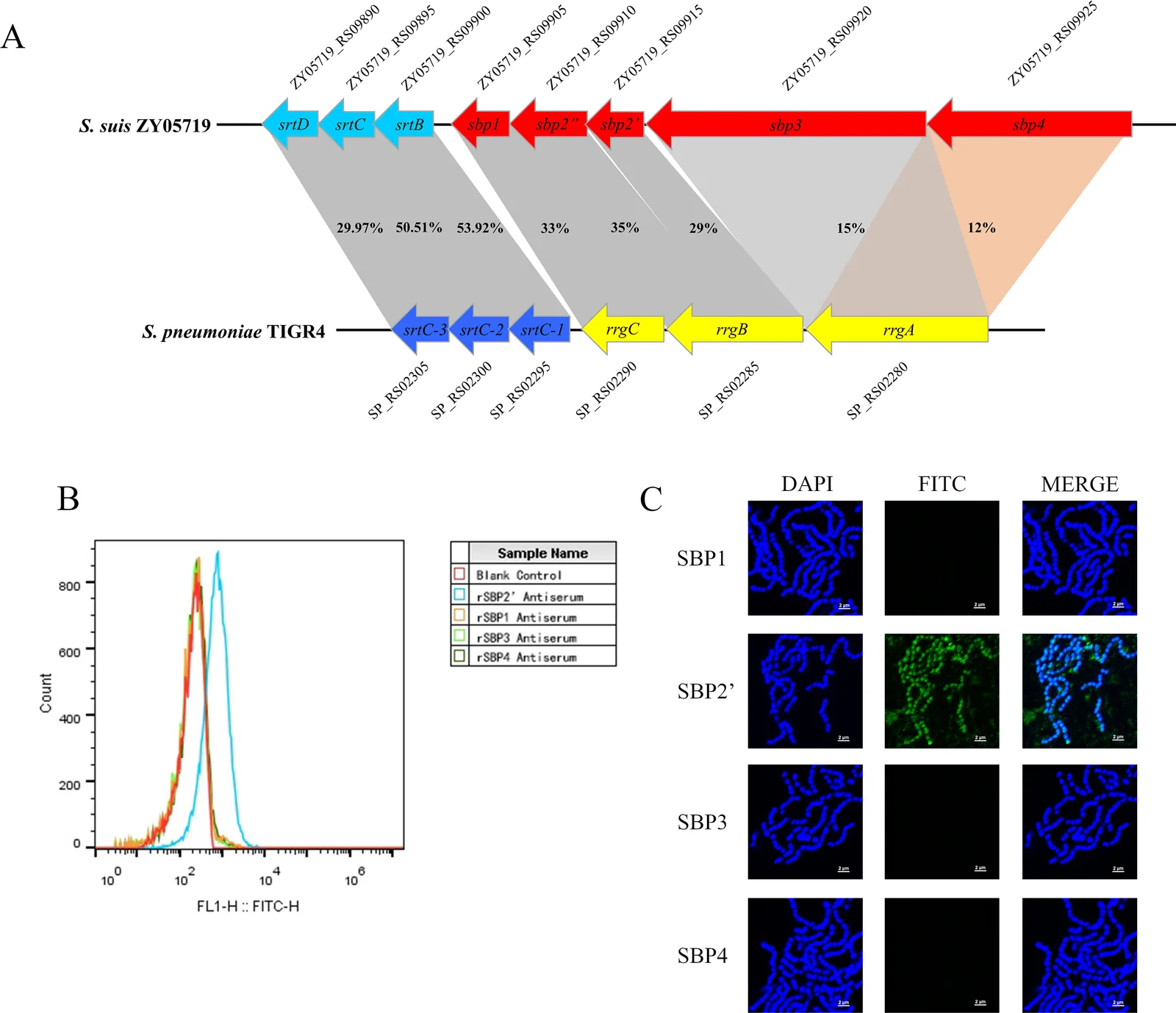
Sequence analysis and subcellular localization of pilus subunits of the *srtBCD* pilus gene cluster. (**A**) Schematic comparison of the *srtBCD* pilus cluster in *S. suis* and the *rlrA* pilus cluster in *S. pneumoniae*. Numbers indicate amino acid sequence identity percentages. Gene colors represent functional categories: light blue, sortases in the *srtBCD* cluster; dark blue, sortases in the *rlrA* cluster; red, pilus subunits in the *srtBCD* cluster; yellow, pilus subunits in the *rlrA* cluster. (**B**) Flow cytometric analysis of SBP2’ surface localization in *S. suis* among pilus subunits. A fluorescence peak shift was observed only for SBP2’ compared with the negative control, whereas SBP1, SBP3, and SBP4 overlapped with the negative control, indicating no detectable surface expression. (**C**) IFA showing surface localization of pilus subunits in *S. suis*. Only SBP2’ exhibited a detectable FITC fluorescence signal on the bacterial surface, while SBP1, SBP3, and SBP4 showed no observable signal.

Sequence alignment revealed high collinearity and conserved gene organization between the *rlrA* and *srtBCD* clusters (Fig. 1A). Both clusters contain three class C sortases responsible for assembling pilus subunits. Amino acid sequence identity analysis showed that the sortases SrtB, SrtC, and SrtD share 53.92%, 50.51%, and 29.97% identity with their respective homologs SrtC-1, SrtC-2, and SrtC-3 in the *rlrA* cluster. Among the pilin subunits, SBP1 showed 33% identity with RrgC, SBP2’ showed 29% identity with the N-terminal region of RrgB, and SBP2’’ showed 35% identity with the C-terminal region of RrgB. In contrast, SBP3 and SBP4 exhibited lower similarity to RrgA, with 15% and 12% identity, respectively.

Both gene clusters encode three class C sortases responsible for pilus assembly (Fig. 1A). The *srtBCD* cluster encodes five pilus subunits, named SBP1, SBP2’, SBP2’’, SBP3, and SBP4, whereas the rlrA cluster encodes the canonical pilus components RrgA, RrgB, and RrgC. The major pilin subunit, SBP2, is truncated into SBP2’ and SBP2’’, which correspond to the N-terminal and C-terminal portions of the RrgB-like major pilin subunit, respectively (Fig. 1A). Sequence motif analysis showed that SBP2’ contains a pilin motif (YPKN) (Fig. S1A), while SBP2’’ contains an E-box motif, which is also a pilin motif, and an LPXTG-like cell wall sorting signal (Fig. S1B). The other minor pilin subunits also contain LPXTG-like motifs (Fig. S1C through E).

Although pilus-associated genes are present, no intact pilus structures were observed on the surface of *S. suis* serotype 2. Flow cytometry analysis demonstrated that only SBP2’ exhibited a fluorescence peak shift compared with the negative control, whereas SBP1, SBP3, and SBP4 showed fluorescence profiles overlapping with the negative control, indicating no detectable surface localization (Fig. 1B). Consistently, IFA revealed that only SBP2’ produced a clear FITC fluorescence signal on the bacterial surface, while the other pilin subunits were not detectable (Fig. 1C).

### SBP2’ is the key component of the *srtBCD* pilus cluster involved in *S. suis* virulence

**Fig 2:**
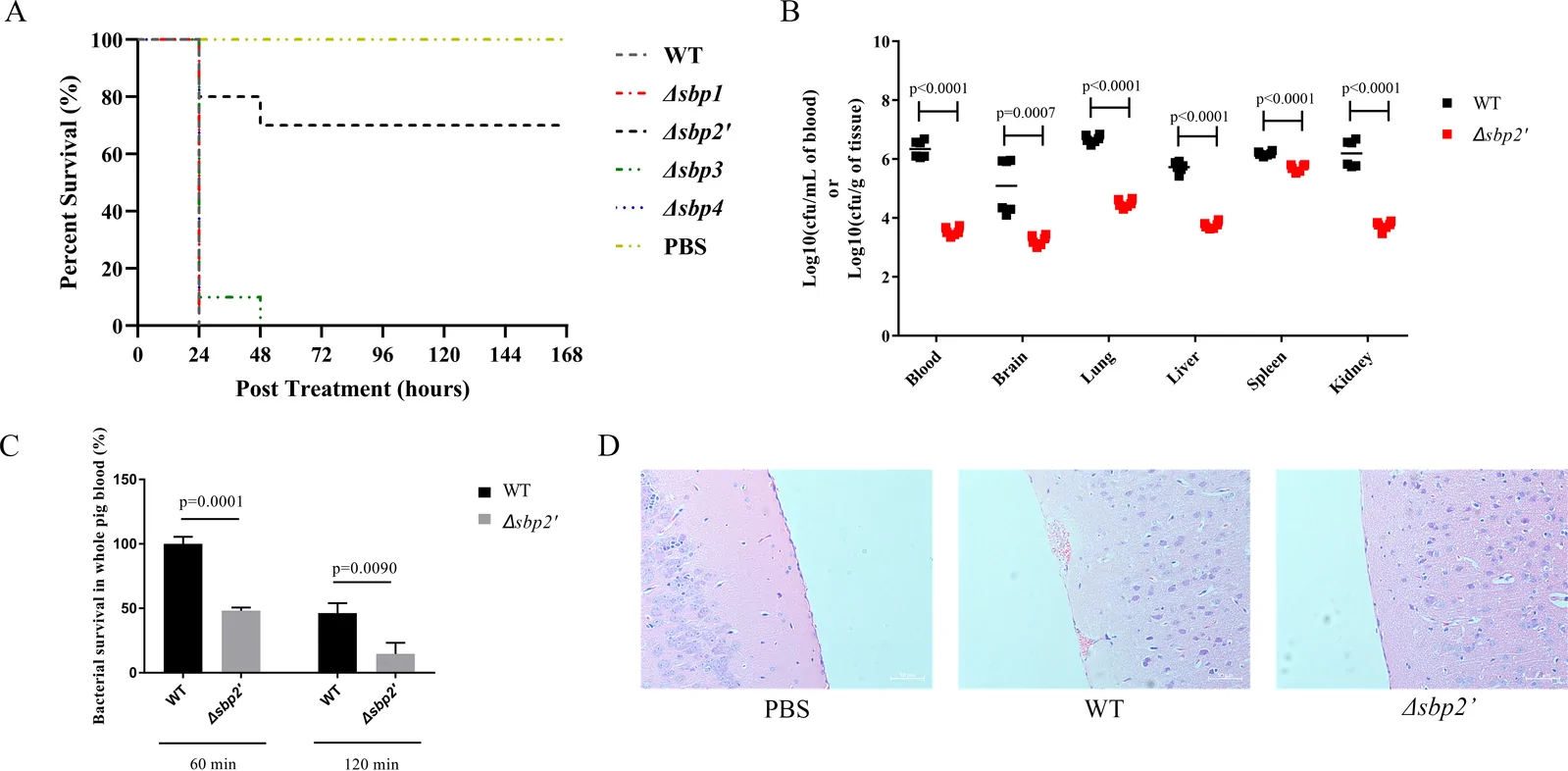
SBP2’ contributes to *S. suis* virulence and brain damage. (**A**) Survival rates of BALB/c mice intraperitoneally challenged with 2 × 10^8^ CFU of WT or mutant strains. Survival curves were analyzed using the log-rank (Mantel-Cox) test. Among all groups, only the *Δsbp2’* mutant showed a statistically significant difference compared with the WT group (*P* = 0.0004). (**B**) Bacterial burdens in the brain, lungs, spleen, and blood at 6 h post-infection. (**C**) Survival of WT and *Δsbp2’* strains in whole pig blood. (**D**) Histopathological analysis of brain tissues from mice infected with WT or *Δsbp2’* strains by H&E staining.

To determine the role of the *srtBCD* pilus gene cluster in *S. suis* virulence, deletion mutants for each pilus subunit were constructed. Following intraperitoneal challenge of BALB/c mice, only the *Δsbp2’* mutant exhibited a significant reduction in virulence compared with the WT strain (*P* = 0.004; Fig. 2A). In contrast, deletion of *sbp1*, *sbp3*, or *sbp4* did not significantly affect virulence (Fig. 2A). Therefore, subsequent bacterial burden assays were performed only with the *Δsbp2’* mutant. Bacterial loads in internal organs and blood were significantly decreased in mice infected with *Δsbp2’* compared with the WT strain (*P* = 0.0007 for the brain and *P* < 0.0001 for the others; Fig. 2B). Similarly, *in vitro* survival assays in whole pig blood demonstrated that the *Δsbp2’* mutant had markedly reduced survival compared with WT (*P* = 0.0001 at 60 min and *P* = 0.0090 at 120 min; Fig. 2C).

Given that meningitis is the most prominent manifestation of *S. suis* infection, we evaluated histopathological changes in mouse brains. Histopathological analysis of brain tissues using H&E staining confirmed the presence of meningeal inflammation in WT-infected mice, which was markedly attenuated in mice infected with *Δsbp2’* (Fig. 2D).

### SBP2’ functions as a host plasminogen receptor in *S. suis*

**Fig 3:**
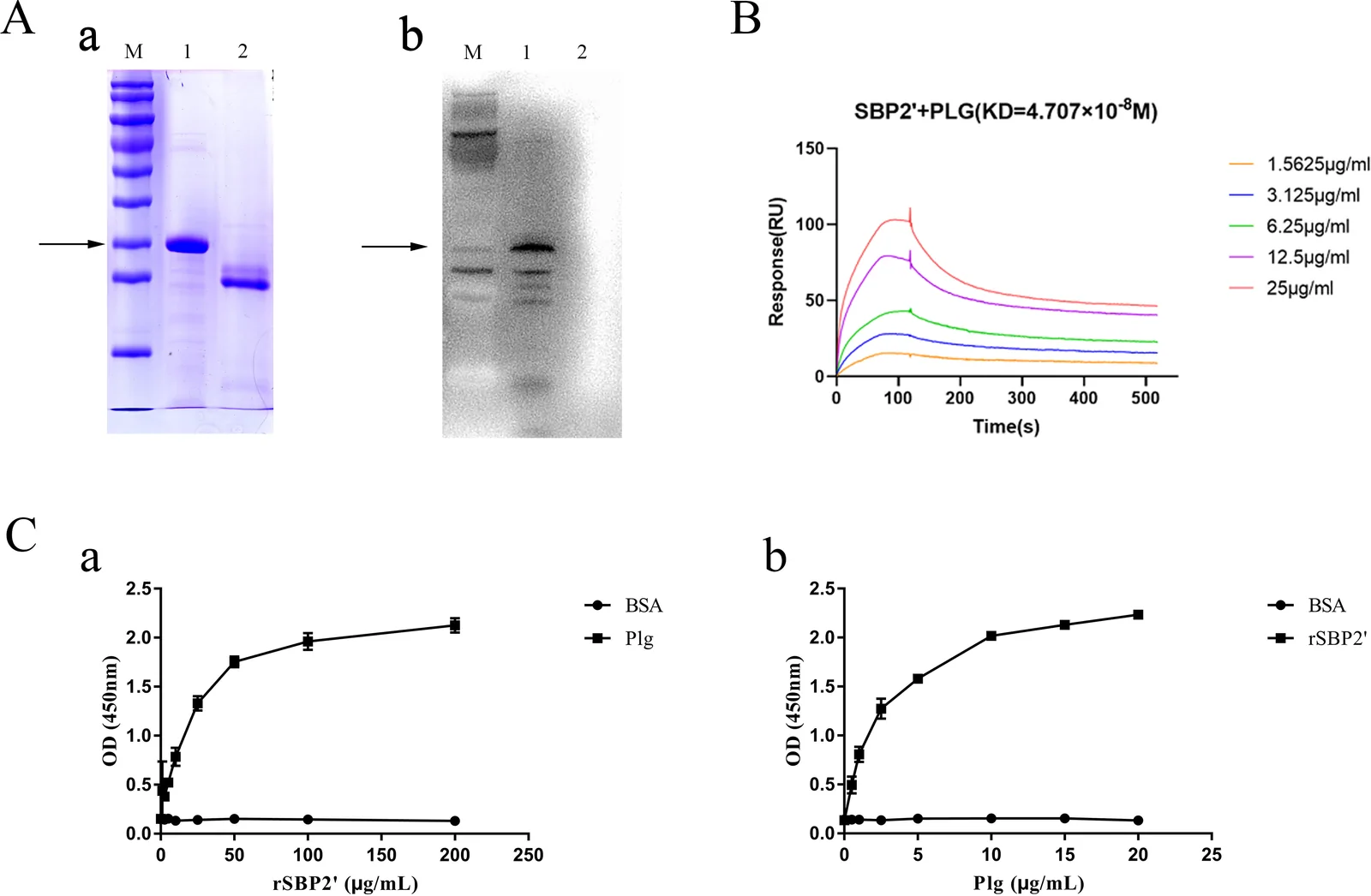
Binding activity of rSBP2’ to host plasminogen. (**A**) Far-Western blot analysis of rSBP2’ binding to host plasminogen. rSBP2’ (lane 1) and a negative control protein rHP07325 (lane 2) were separated by SDS-PAGE. (**Aa**) One gel was stained with Coomassie Blue G-250. (**Ab**) Another gel was transferred to a PVDF membrane for Far-Western blot; exposure time: 5 s. (**B**) Surface plasmon resonance sensorgrams depicting the binding of increasing concentrations of rSBP2’ to immobilized plasminogen. Flow rate: 30 μL/min for 180 s. RU, resonance units. (**Ca, Cb**) Dose-dependent binding between rSBP2’ and plasminogen measured by ELISA. Experiments were performed independently three times

Far-Western blotting was performed to assess the binding of rSBP2’ to host plasminogen. A band corresponding to the purified rSBP2’ was detected, indicating specific binding (Fig. 3A). High-affinity interactions were further confirmed by surface plasmon resonance (Fig. 3B). ELISA-based binding assays demonstrated that soluble plasminogen bound to immobilized rSBP2’ in a concentration-dependent manner, as indicated by the gradual increase in absorbance at 450 nm with increasing plasminogen concentrations. Reciprocal experiments using immobilized plasminogen and soluble rSBP2’ yielded similar results. In addition, rSBP2’ also exhibited dose-dependent binding to fibronectin, further supporting its role as a host protein-binding factor (Fig. 3C).

### SBP2’ binds to hBMECs and interacts with the extracellular matrix

**Fig 4:**
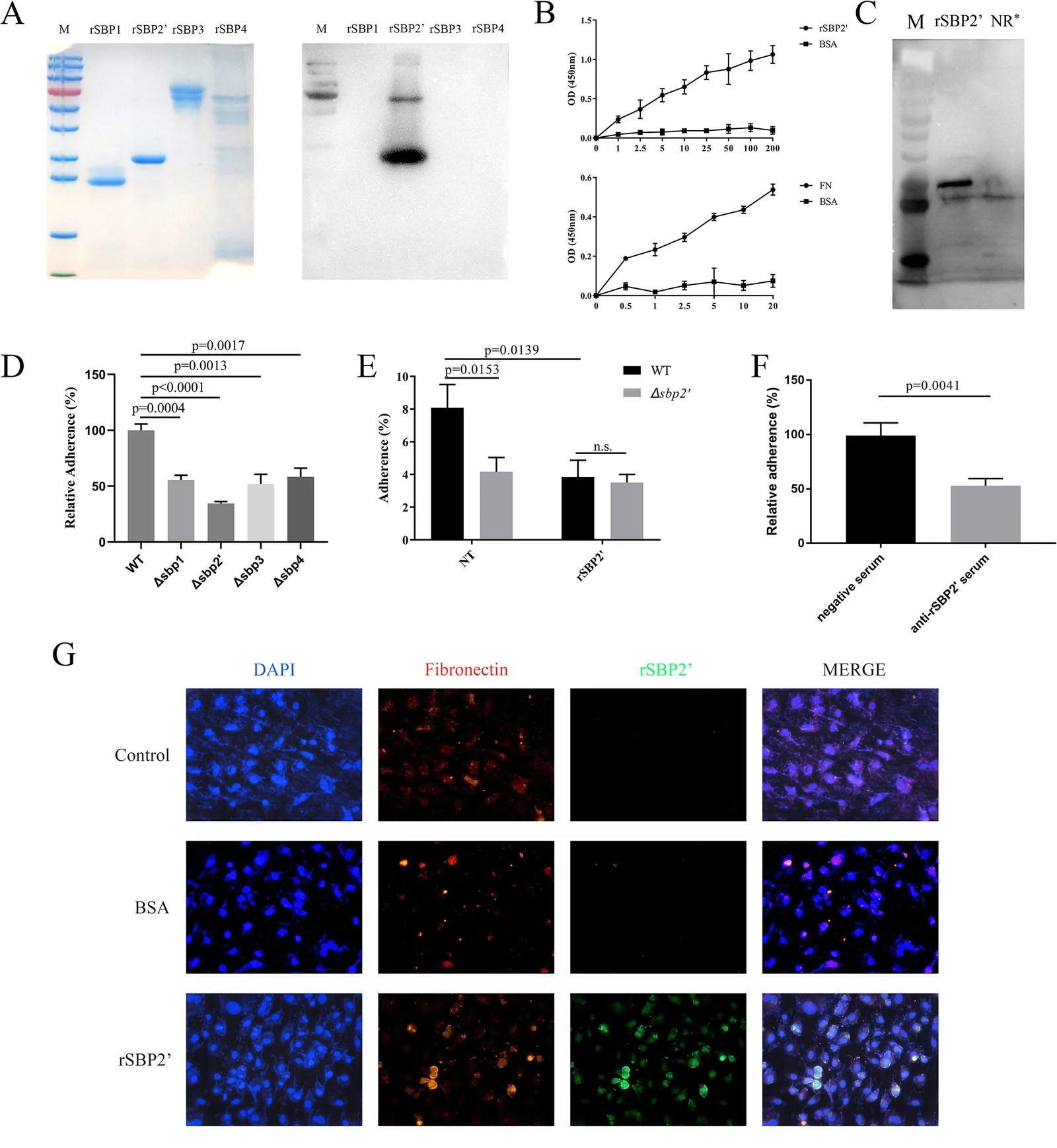
SBP2’ mediates the initial colonization of *S. suis* on hBMEC cells. (**A**) Determination of fibronectin (FN)-binding ability of srtBCD pilus subunits by Far-Western blot. (Left) Coomassie G-250-stained gel. (Right) Far-Western blot analysis. (**B**) ELISA-based detection of rSBP2’ binding to fibronectin. Microtiter plates were coated with either (top) rSBP2′ (5 μg/mL) or (bottom) fibronectin (5 μg/mL), followed by incubation with increasing amounts of fibronectin or rSBP2’. (**C**) Protein binding assay with hBMEC cells. Cells were digested, collected, and incubated with 5 μg rSBP2’. NR indicates non-relevant protein HP07325. (**D**) Relative adherence rate of WT and mutant strains in hBMEC cells. (**E**) Pre-treatment of hBMEC cells with 10 μg rSBP2’ inhibited *S. suis* adherence. (**F**) Pre-treatment of bacteria with anti-SBP2’ antiserum reduced adherence to hBMEC cells. (**G**) Co-localization of SBP2’ and fibronectin in hBMEC cells observed by immunofluorescence.

We further investigated the interaction between the *srtBCD* pilus cluster and host components. Considering the importance of the ECM in host-pathogen interactions, and with FN being a major ECM component, the binding of pilus subunits to FN was evaluated. Among the srtBCD subunits, only SBP2’ specifically bound to FN (Fig. 4A). ELISA analysis confirmed dose-dependent binding of soluble FN to immobilized SBP2’, and reciprocal experiments yielded similar results, indicating high-affinity interactions (Fig. 4B).

The role of the *srtBCD* pilus cluster in mediating interactions with hBMECs was examined to evaluate its potential in promoting *S. suis* transit across the BBB. Deletion of *sbp2’* significantly reduced bacterial adherence to hBMECs (*P* < 0.0001; Fig. 4C and D). Meanwhile, deletion of the three minor pilus subunits showed similar trends, although less pronounced (*P* = 0.0004 for *Δsbp1*, *P* = 0.0013 for *Δsbp3*, and *P* = 0.0017 for *Δsbp4*; Fig. 4D). In addition, IFA was performed by incubating hBMECs with recombinant pilus proteins followed by antibody detection. Fluorescence signals corresponding to all four pilus subunits were observed on the surface of hBMECs, indicating that each pilus protein is capable of binding to hBMECs (Fig. S2).

Furthermore, adherence inhibition assays using anti-SBP2’ serum and recombinant SBP2’ as inhibitors demonstrated that all these treatments blocked *S. suis* adherence to hBMECs, confirming the critical roles of SBP2’ and FN in mediating interactions between *S. suis* and the BBB (*P* = 0.0139 for rSBP2’ treatment; Fig. 4E, *P* = 0.0041 for anti-SBP2’ serum treatment; Fig. 4F). Co-localization of rSBP2’ and FN on hBMECs was visualized by IFA (Fig. 4G). Collectively, these data suggest that SBP2’ contributes to the colonization of *S. suis* on the BBB.

### SBP2’ mediates host ECM degradation by hijacking host plasminogen

**Fig 5:**
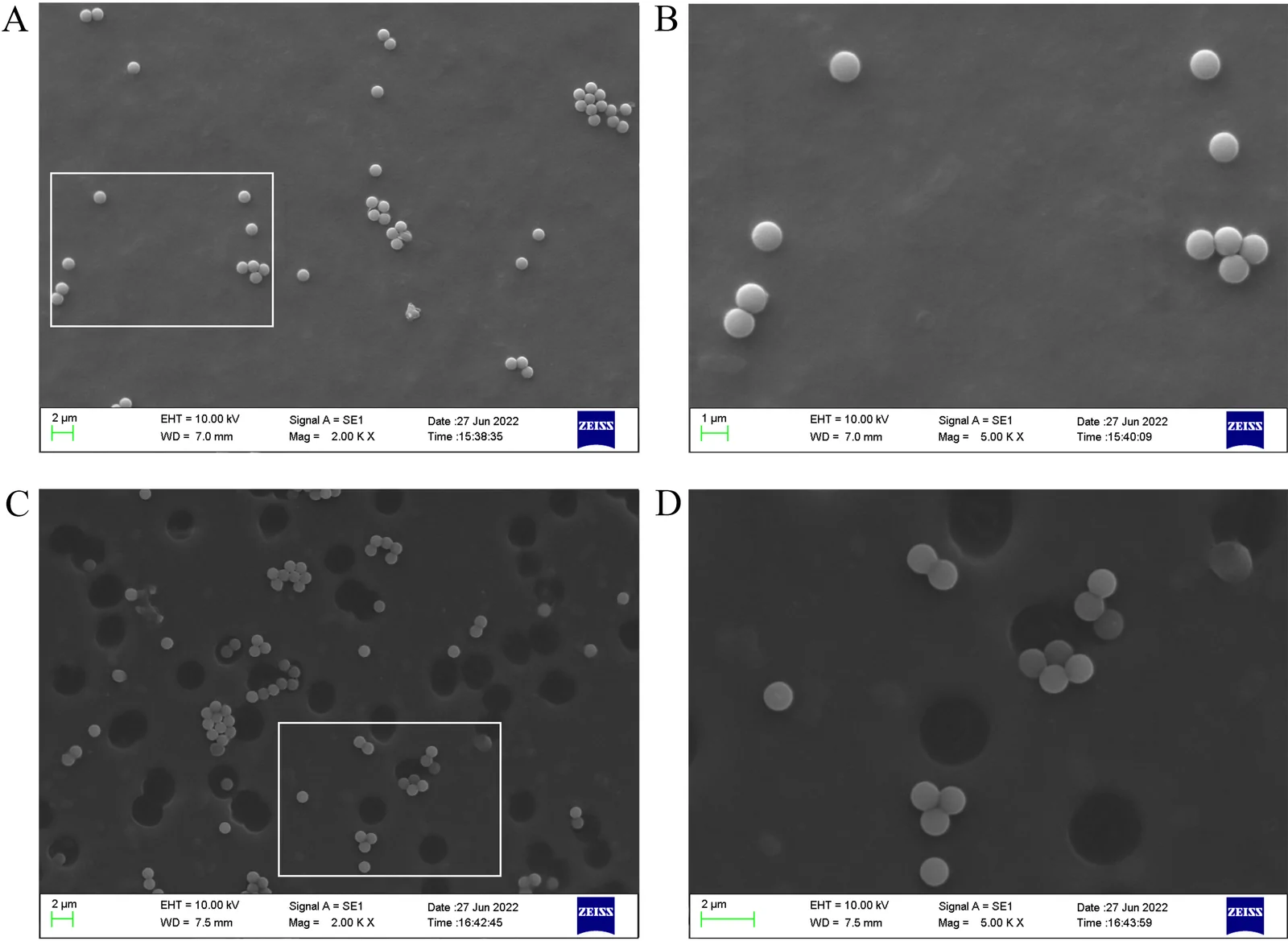
Electron microscopic visualization of Matrigel-reconstituted basement membrane degradation. BSA-coated control beads (**A, B**) or rSBP2’-coated polystyrene beads (**C, D**) were pre-incubated with plasminogen and tPA, then added to 3-μm Transwell filters coated with Matrigel-reconstituted basement membrane. After 40 h incubation, filters were fixed with 2.5% glutaraldehyde and examined by scanning electron microscopy. Panels B and D show enlarged views of portions of panels A and C, respectively.

The ECM is a critical physical barrier against bacterial infection in host tissues. Plasmin-mediated ECM degradation may facilitate bacterial penetration of host tissue barriers. To evaluate whether rSBP2’ facilitates protease-dependent degradation of the ECM, a Matrigel-based Transwell system was used as an *in vitro* ECM model. Scanning electron microscopy revealed that the ECM structure remained largely intact in the BSA-coated control group, with a smooth and continuous matrix surface. In contrast, the rSBP2’-coated bead group induced pronounced disruption of the Matrigel architecture, characterized by extensive surface degradation, visible perforations, and beads embedded within the matrix. These results suggest that rSBP2’ enhances plasminogen-dependent ECM degradation, thereby promoting structural breakdown of basement membrane-like barriers (Fig. 5).

### SBP2’ contributes to anti-phagocytosis of *S. suis* in the brain

**Fig 6:**
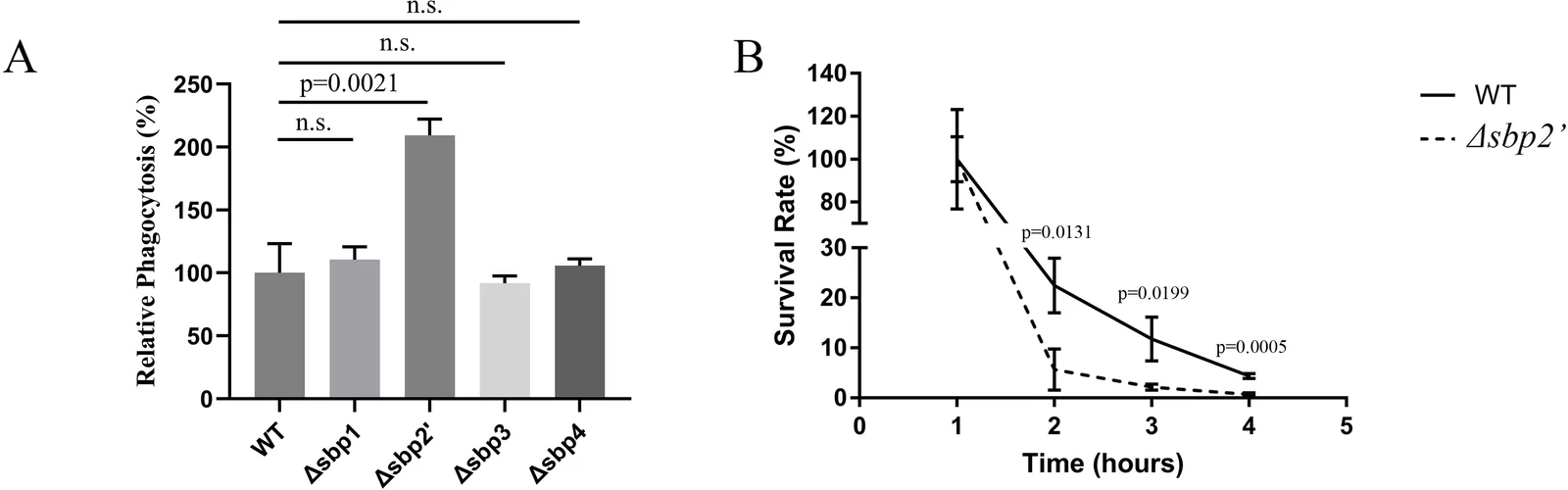
SBP2’ mediates anti-phagocytosis of *S. suis* in BV-2 cells. (**A**) Relative phagocytosis rate of WT and mutant strains in BV-2 cells. (**B**) Relative intracellular survival of *S. suis* over time in BV-2 cells. ns, not significant.

After crossing the endothelial barrier, *S. suis* can be captured by macrophages, and microglia serve as the resident macrophages in brain parenchyma. The role of SBP2’ in anti-phagocytosis was evaluated using BV2 cells. Upon incubation, the number of intracellular bacteria in the *Δsbp2’* group was significantly higher than in the WT group (*P* = 0.0021; Fig. 6A). By setting the initial intracellular bacterial load as 100%, the relative survival of bacteria inside BV2 cells was monitored at 1, 2, and 3 h. The *Δsbp2’* mutant exhibited significantly lower intracellular survival compared with WT (*P* = 0.0131 for 2 h, *P* = 0.0199 for 3 h, and *P* = 0.0005 for 4 h; Fig. 6B). Deletion of the three minor pilus subunits did not affect anti-phagocytic ability (Fig. 6A). These results suggest that SBP2’ plays a critical role in the anti-phagocytic capacity and intracellular survival of *S. suis*.

### Protective role of SBP2’ monoclonal antibody

**Fig 7:**
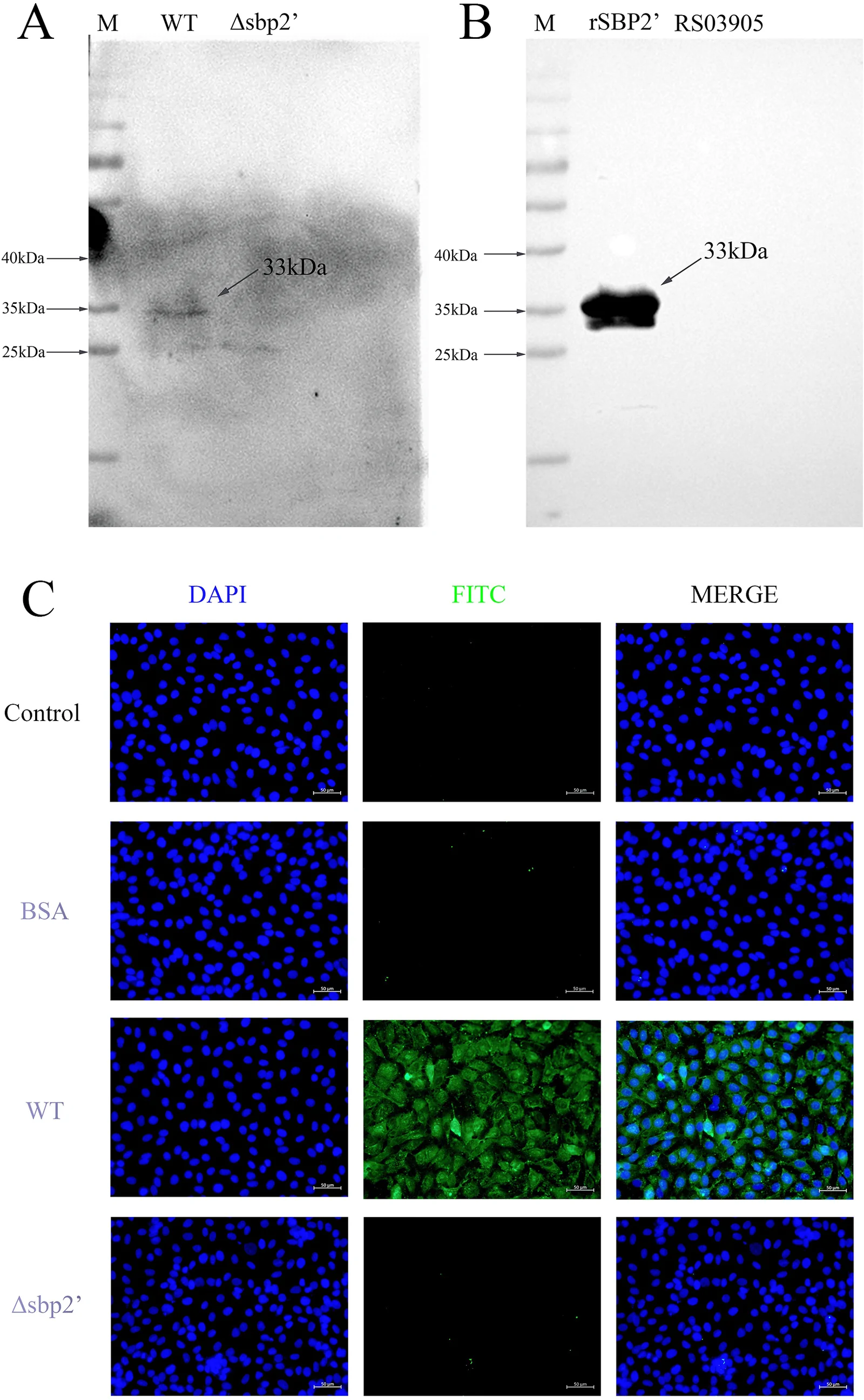
Specificity testing of SBP2’ monoclonal antibody. (**A**) Western blot detection of SBP2’ in WT and *Δsbp2’* strains using SBP2’ mAb. (**B**) Western blot analysis confirming the specificity of SBP2’ mAb against rSBP2’. (**C**) Immunofluorescence showing specific detection of *S. suis* adhered to hBMEC cells by SBP2’ mAb.

Monoclonal antibodies (mAbs) against SBP2’ were generated, and the isotype was determined to be IgG2b/κ. The specificity of the mAb was confirmed by Western blot, showing a distinct band in the WT strain that was absent in the *Δsbp2’* mutant (Fig. 7A). Recombinant SBP2’ protein also reacted specifically with the mAb (Fig. 7B). Immunofluorescence assays demonstrated green fluorescence on hBMECs incubated with WT *S. suis* and detected by SBP2’ mAb, while no signal was observed with *Δsbp2’* (Fig. 7C).

**Fig 8:**
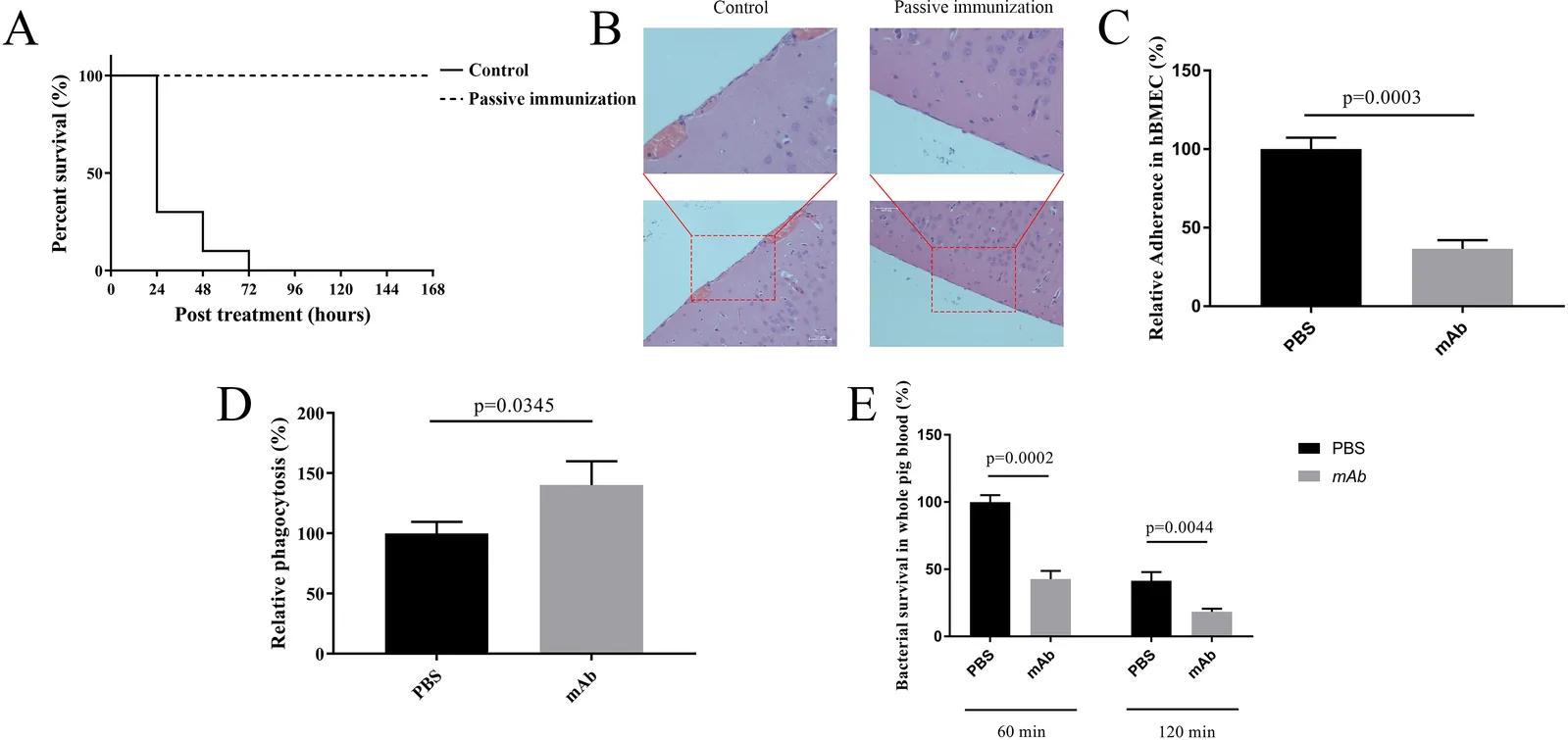
Protective effects of SBP2’ monoclonal antibody against *S. suis* infection. (**A**) Survival rates of mice pre-injected with SBP2’ mAb and subsequently challenged with 2 × 10^8^ CFU/mouse of *S. suis* serotype 2. (**B**) Histopathological assessment of mouse brains after immunization with SBP2’ mAb or PBS and subsequent infection with *S. suis* serotype 2. (**C**) Inhibition of *S. suis* adherence to hBMEC cells by pre-treatment with SBP2’ mAb. (**D**) Enhancement of phagocytosis of *S. suis* by BV-2 cells after pre-treatment with SBP2’ mAb. (**E**) Survival percentage of *S. suis* in whole pig blood following pre-treatment with SBP2’ mAb or PBS.

Passive immunization with SBP2’ mAb conferred protection to ICR mice against *S. suis* infection (Fig. 8A). Brain pathology was alleviated in immunized mice, with reduced hyperemia compared with control mice (Fig. 8B). Pre-incubation of *S. suis* with SBP2’ mAb significantly reduced bacterial adherence to hBMECs (*P* = 0.0003; Fig. 8C). *In vitro* opsonophagocytosis assays showed enhanced phagocytosis of *S. suis* by BV2 cells following pre-treatment with SBP2’ mAb (*P* = 0.0345; Fig. 8D). Additionally, bacterial survival in whole pig blood was significantly reduced when *S. suis* was pre-treated with SBP2’ mAb (*P* = 0.0002 at 60 min and *P* = 0.0044 at 120 min; Fig. 8E).

Collectively, these results indicate that SBP2’ mAb provides effective immunoprotection against *S. suis* infection and can inhibit bacterial adherence, phagocytosis resistance, and survival in blood, highlighting its potential utility in controlling infection.

## DISCUSSION

Pili play crucial roles in the initial colonization of host tissues, interaction with host cells, modulation of immune responses, and the progression to invasive disease in both Gram-negative and Gram-positive bacteria. In *N. meningitidis*, type IV pili have been reported as essential adhesins that contribute to brain invasion through activation of the CD147–β2AR complex (36, 37). In *Streptococcus agalactiae*, the ancillary pilus subunit PilA mediates adherence to brain endothelium and activates the FAK signaling pathway via binding to collagen and α2β1 integrins (38). The *rlrA* pilus gene cluster, homologous to the *srtBCD* cluster, facilitates translocation from the bloodstream across the BBB to the brain, causing lethal meningitis in *S. pneumoniae* (39). Furthermore, pili have been shown to mediate host specificity, such as nonsynonymous single-nucleotide polymorphisms in FimH, which modulate the adhesion preference of *Salmonella enterica* serovar Typhimurium to different host cells (18).

Meningitis is a hallmark of *S. suis* infection, and extensive research has focused on elucidating its underlying mechanisms. Several meningitis-associated virulence factors have been identified. MRP, a major virulence determinant, can bind fibrinogen and disrupt p120-catenin in brain endothelial cells (11). Enolase interacts with 40S ribosomal protein SA on the surface of hBMECs and induces apoptosis, contributing to meningitis (12). SspepO binds multiple types of ECM, facilitating disease progression (13, 40). However, some virulent *S. suis* serotype 2 strains lacking MRP can still cause severe disease (41, 42), and Enolase and SspepO are ubiquitous among both virulent and avirulent strains (7). These observations suggest that additional genes or proteins may play more critical roles in the formation of meningitis by *S. suis*.

Previously, Shao et al. reported that deletion of *sbp2’* led to reduced virulence of *S. suis* in a zebrafish model (43). Comparative proteomic analysis using two-dimensional difference gel electrophoresis on two artificially derived *S. suis* mutants with differing virulence demonstrated that SBP2’ was differentially expressed and played a central role in the *S. suis* virulence factor interaction network (20). Genomic analyses of subsets of *S. suis* serotype 2 isolates with varying virulence further revealed that the *srtBCD* pilus gene cluster is associated with virulence and may serve as a potential virulence marker (21, 23). In this study, we evaluated the role of individual pilin subunits within the *srtBCD* cluster in mediating *S. suis* virulence. Although all four pilin subunits contributed to bacterial adhesion to host cells, only deletion of *sbp2’* significantly reduced virulence. This finding indicates that SBP2’ is the key constituent responsible for virulence within the *srtBCD* pilus gene cluster.

Several cell surface-expressed plasminogen receptors have been well characterized, including in *S. suis* (40, 44). Typically, these receptors possess an N-terminal signal sequence and a membrane anchor motif. In the case of the truncated major pilin subunit, the E-box and LPXTG-like motifs are located in the SBP2'' fragment, raising questions about the subcellular localization of SBP2’. Our analyses showed that SBP2’ can still be detected on the cell surface of *S. suis*. However, the mechanism underlying its localization remains unknown, and it cannot yet be conclusively stated that SBP2’ is anchored to the cell surface.

After recruitment of host plasminogen to the bacterial cell surface via plasminogen receptors, plasminogen can facilitate bacterial invasion by degrading the ECM (24). Having identified rSBP2’ as a plasminogen receptor, we further found that SBP2’ also binds to fibronectin, a major component of host ECM. *In vitro* assays confirmed the role of SBP2’ in the initial colonization of *S. suis*, and co-localization of rSBP2’ and fibronectin was observed. Given that SBP2’ recruits host plasminogen, the co-localization of plasmin and ECM constituents likely promotes ECM degradation, which was further verified experimentally. Disruption of the ECM facilitates bacterial penetration of the BBB. These results are consistent with studies on streptococcal pili, including the minor pilin subunit Cpa in *Streptococcus pyogenes*, PilA in *Streptococcus agalactiae*, and RrgA in *S. pneumoniae*, all of which bind various ECM proteins such as fibronectin, collagen, and laminin, and contribute to bacterial virulence (38, 45, 46). Collectively, these findings indicate that SBP2’ is a novel plasminogen receptor in *S. suis* that mediates the first step of host colonization and promotes BBB penetration by recruiting host plasminogen to the bacterial cell surface.

BMECs are the primary cellular component of the BBB (47). After crossing the endothelium, bacteria are captured by microglia, the resident macrophages in the brain parenchyma. Bacterial components, particularly pathogen-associated molecular patterns, activate microglia and stimulate the release of pro-inflammatory factors (48). In this study, we found that SBP2’ significantly enhances the anti-phagocytic ability of *S. suis*. Moreover, the intracellular survival of *S. suis* within microglia was affected by SBP2’: the *sbp2’* deletion mutant was cleared more rapidly from microglia compared with the wild-type strain. These findings suggest that SBP2’ is important for intracellular survival, providing *S. suis* time to evade host immune responses and establish infection within the central nervous system.

Pilus subunits have long been considered promising subunit vaccine candidates (49). In many bacteria, including *Acinetobacter baumannii* (50), *Escherichia coli* (51), and also *Streptococcus* species (52), their immunogenicity and protective efficacy have been demonstrated. Previously, our group reported that rSBP2’ could confer robust immunoprotection against *S. suis* infection in an ICR mouse model (53). With advances in genetically engineered antibodies and the rise of antimicrobial resistance, the therapeutic potential of mAbs has increased rapidly (54). Although bacterial exotoxins have traditionally been the primary targets for mAbs, emerging evidence indicates that mAbs targeting bacterial surface proteins can also mediate antibacterial effects (55). Importantly, some surface proteins are conserved among clinical isolates and may be suitable for prophylactic or therapeutic intervention (56). For example, in *Pseudomonas aeruginosa*, antibodies targeting the pilus protein PcrV have been shown to treat acute pneumonia and reduce organ bacterial burden (57). Considering the zoonotic nature of *S. suis*, development of effective mAbs is highly valuable. In this study, we generated an mAb targeting SBP2’, which conferred immunoprotection against *S. suis* infection in ICR mice through passive immunization. *In vitro* assays further demonstrated that SBP2’ mAb enhances opsonophagocytosis and blocks bacterial adhesion to host cells. Collectively, these data suggest that SBP2’ represents a promising target for monoclonal antibody-based therapy against *S. suis*.

Although multiple serotypes have been reported in human *S. suis* infections, virulent serotype 2 is the most commonly associated with human disease (8, 58), followed by serotype 14 (59, 60), which is phylogenetically close to virulent serotype 2. Only sporadic cases of other serotypes have been reported (61–63). Interestingly, we found that *sbp2’* is present only in serotype 14 isolates (regardless of virulence status) and virulent serotype 2 isolates. Notably, the *srtBCD* cluster is more widely distributed among CC1 strains than among other clonal complexes, and CC1 strains are more frequently associated with meningitis and invasive infections in humans. This association suggests that the *srtBCD* cluster may contribute to the enhanced neuroinvasive potential of specific *S. suis* lineages. This suggests that *sbp2’* may serve as a potential marker for human infection, and monoclonal antibodies against SBP2’ could be developed into diagnostic tools. However, not all CC1 strains necessarily harbor the *srtBCD* cluster, indicating substantial genetic diversity even within this clonal complex. In addition, meningitis caused by strains belonging to other CCs may involve distinct pathogenic mechanisms that do not rely on the SBP2’-associated pathway. Previous studies have also demonstrated that *S. suis* undergoes extensive transcriptional reprogramming in cerebrospinal fluid compared with standard culture conditions, with numerous virulence- and metabolism-associated genes showing altered expression. These observations suggest that the development of meningitis is likely a multifactorial process involving coordinated regulation of multiple genes and pathways, rather than being driven by a single virulence determinant alone (64).

Despite the significant insights provided by this study, several limitations should be acknowledged. First, our mechanistic evaluations of SBP2’ in mediating BBB traversal, such as cell adhesion, invasion, and ECM degradation, were predominantly conducted using *in vitro* cell lines (hBMECs and BV2 cells). Although these cell lines are well-established models, they cannot fully replicate the complex physiological microenvironment and cellular cross-talk of the *in vivo* BBB. Second, while the SBP2’ monoclonal antibody demonstrated promising immunoprotective efficacy in the murine model, mice are not the natural host for *S. suis*. Therefore, the translational potential of these findings requires further validation.

Future perspectives should focus on utilizing more physiologically relevant models, such as 3D organ-on-a-chip BBB systems or natural host (pig) infection models, to capture the full spectrum of *S. suis* pathogenesis. Additionally, further studies are warranted to investigate the detailed downstream signaling pathways triggered by the SBP2’-plasminogen interaction and to evaluate the clinical efficacy of SBP2’-targeted therapeutic interventions or subunit vaccines in swine.

Overall, this study demonstrates that the truncated major pilin subunit SBP2’ of the *srtBCD* cluster mediates colonization of brain endothelial cells through interaction with host plasminogen and contributes to intracellular survival within microglia. Moreover, passive immunization with SBP2’ monoclonal antibody effectively protects mice from *S. suis* infection. Taken together, our findings provide new insights into the role of pilus components in the pathogenesis of *S. suis*.

## Data Availability

The data sets generated and/or analyzed during the current study are available in the GenBank repository under accession numbers NZ_CP007497.1 for ZY05719 and WP_012027978.1 for SBP2’.

## SUPPLEMENTAL MATERIAL

Supplemental figures aem.01267-26-s0001.docx Fig. S1 and S2.

Table S1 aem.01267-26-s0002.xlsx Characteristics of the strains, plasmids, and primers used in this study.

ASM does not own the copyrights to Supplemental Material that may be linked to, or accessed through, an article. The authors have granted ASM a non-exclusive, world-wide license to publish the Supplemental Material files. Please contact the corresponding author directly for reuse.
