# Supplementary material for: The truncated major pilin subunit SBP2’ contributes to Streptococcus suis meningitis by interacting with host plasminogen
Source: Appl Environ Microbiol. 2026 Aug 10;92(9):e01267-26. doi: 10.1128/aem.01267-26 (PMC13599487; doi:10.1128/aem.01267-26)
Supplement: Supplementary file 1 [file aem.01267-26-s0001.docx]

Supplementary Figure. 1 Amino acid sequence alignment of pilin subunits in the *srtBCD* pilus gene cluster and the *rlrA* pilus gene cluster. A) SBP2’; B) SBP2’’; C) SBP1; D) SBP3; E) SBP4

Supplementary Figure. 2 Specific binding of pilin subunits to hBMEC cells assessed by immunofluorescence.
